# Supplementary material for: “Everything had stopped, no meeting, no gathering”: Social interactions during the COVID-19 pandemic in the Central African Republic, the Democratic Republic of Congo, and Bangladesh
Source: PLoS One. 2025 May 27;20(5):e0323108. doi: 10.1371/journal.pone.0323108 (PMC12112413; doi:10.1371/journal.pone.0323108)
Supplement: S1 File — (DOCX) [file pone.0323108.s001.docx]

Supplementary material

Table of Contents

[1 Method 1](#_Toc170301796)

[1.1 FGD participants profiles 1](#_Toc170301797)

[1.2 Aggregated score for knowledge of COVID 3](#_Toc170301798)

[2 Results 4](#_Toc170301799)

[2.1 Respondents’ profile 5](#_Toc170301800)

# Method

## FGD participants profiles

Table S1: Profile of Key Informants, Cox’s Bazar district, Bangladesh

| **Age** | **Gender** | **Location** | **Location Type** |
| --- | --- | --- | --- |
| 20-29 | F | Kutubdia | Rural |
| 20-29 | M | Moheshkali | Rural |
| 40-49 | M | Moheshkali | Rural |
| 40-49 | F | Pekua | Rural |
| 40-49 | F | Ukhiya | Rural |
| 20-29 | M | Ukhiya | Rural |
| 30-39 | M | Teknaf | Rural |
| 60-69 | M | Moheshkali | Rural |
| 60-69 | M | Pekua | Rural |
| 30-39 | M | Moheshkali | Rural |
| 30-39 | F | Moheshkali | Rural |
| 60-69 | F | Kutubdia | Rural |
| 20-29 | F | Teknaf | Rural |
| 60-69 | F | Teknaf | Rural |
| 20-29 | M | Pekua | Rural |
| 60-69 | M | Ukhiya | Rural |
| 30-39 | M | CXB Sadar | Rural |
| 20-29 | F | CXB Sadar | Urban |
| 50-59 | F | CXB Sadar | Urban |
| 40-49 | M | Kutubdia | Urban |
| 20-29 | F | Chakaria | Rural |
| 40-49 | F | Chakaria | Rural |
| 40-49 | M | Chakaria | Rural |

Table S2: Characteristics of focus group discussions' participants, Mweso health zone, DRC

| **FGD #** | **Sex** | **Number of participants** | **Age category** | **Health zone** | **Settlement** |
| --- | --- | --- | --- | --- | --- |
| **FGD 1** | Women | 8 | 31 – 59 | Mweso | K |
| **FGD 2** | Men | 8 | 18 – 30 | Mweso | K |
| **FGD 3** | Men | 8 | 60 + | Mweso | K |
| **FGD 4** | Men | 9 | 18 – 30 | Mweso | B |
| **FGD 5** | Men | 10 | 60 + | Mweso | B |
| **FGD 6** | Men | 10 | 31 – 59 | Mweso | B |
| **FGD 7** | Women | 10 | 31 – 59 | Mweso | Ki |
| **FGD 8** | Women | 9 | 18 – 30 | Mweso | Ki |
| **FGD 9** | Women | 9 | 31 – 50 | Mweso | Ki |
| **FGD 10** | Women | 9 | 18 – 30 | Mweso | M |
| **FGD 11** | Women | 10 | 60 + | Mweso | M |
| **FGD 12** | Men | 10 | 31 – 59 | Mweso | M |

Table S3: Characteristics of focus group discussions' participants, Central African Republic

| Health District | Area Type | Sex | Age / category | Status | # of participants |
| --- | --- | --- | --- | --- | --- |
| Bégoua | Urban | Female | 31-59 | non-displaced | 7 |
| Bégoua | Urban | Male | 18-30, 31-59 | non-displaced | 8 |
| Bégoua | Rural | Mixed | 60+ | non-displaced | 8 |
| Bégoua | Rural | Male | 31-59 | non-displaced | 10 |
| Bégoua | Urban | Female | 31-59 | non-displaced | 7 |
| Bégoua | Urban | Female | 18-31, Pregnant, nursing Female | non-displaced | 7 |
| Bégoua | Rural | Male | 31-59 | non-displaced | 10 |
| Bégoua | Rural | Female | 31-59 | non-displaced | 8 |
| Bimbo | Rural | Male | 18-30 | non-displaced | 9 |
| Bimbo | Rural | Female | 31-59 | non-displaced | 8 |
| Bimbo | Urban | Male | 18-30 | IDPs (site) | 8 |
| Bimbo | Urban | Female | 31-59 | IDPs (site) | 9 |
| Bimbo | Urban | Male | 31-59 | non-displaced | 8 |
| Bimbo | Urban | Female | 18-30 | non-displaced | 9 |
| Bimbo | Urban | Mixed | 60+ | non-displaced | 6 |
| Bimbo | Urban | Mixed | Vulnerable | non-displaced | 4 |
| Bimbo | Urban | Female | 31-59 | IDPs (site) | 8 |
| Bangui | Urban | Female | 18-30 | IDPs (site) | 8 |
| Bangui | Urban | Mixed | 60+ | IDPs (site) | 9 |
| Bangui | Urban | Male | 31-59 | IDPs (site) | 9 |
| Bangui | Urban | Male | 31-59 | non-displaced | 8 |
| Bangui | Urban | Male | 18-30 | non-displaced | 8 |
| Bangui | Urban | Mixed | Vulnerable | non-displaced | 8 |
| Bangui | Urban | Female | 18-30 | non-displaced | 8 |

## Aggregated score for knowledge of COVID

We investigated level of knowledge about COVID-19 at the time of data collection, using three multiple choice questions (table S4). An aggregated score was calculated as the average of the question specific scores and respondents classified as not / partially / informed or well informed.

Table S4: Classification of respondents by knowledge related to key characteristics of COVID-19

| **Question** | **Score** | **Options** |
| --- | --- | --- |
| In your opinion, who is the most susceptible to falling seriously ill due to Coronavirus? | **Well informed**: corresponds to those who selected the 4 correct options | Everyone  Elderly people (60+ years)  Adults (19-59 years)  People with pre-existing conditions (respiratory problems, heart problems, etc) |
|  | **Informed**: corresponds to those who selected 3 out of 4 correct options. |  |
|  | **A little informed**: corresponds to those who selected 1 of these options. | Everyone  Elderly people (60+ years)  Adults (19-59 years)  Children (0-18 years)  People with pre-existing conditions (respiratory problems, heart problems, etc)  Health workers |
|  | **Not at all informed**: corresponds to those who selected other options. | Pregnant or nursing women  Do not know / prefer not to respond |
|  |  |  |
| How can a person contract COVID-19? | **Well informed**: corresponds to those who selected all 3 correct options. | Via particles in the air (when others cough / sneeze)  Via physical contact with infected people  Via physical contact with a contaminated object or surface |
|  | **Informed**: corresponds to those who selected 2 out of 3 correct options. |  |
|  | **A little informed**: corresponds to those who selected 1 out of 3 correct options |  |
|  | **Not at all informed**: corresponds to those who chose only among these other options. | By drinking contaminated water  By washing in contaminated water  By eating certain foods  Contaminated breastmilk / breastfeeding  Other  Do not know / prefer not to respond |
|  |  |  |
| In your opinion, is it possible to take measures to reduce the risk of contracting COVID-19?  If yes, how do you reduce the risk of contracting COVID-19? | **Well informed**: corresponds to those who selected the 6 answers which refer to « preventative measures » | Reduce contact with others by avoiding crowds, staying at home, etc  Increase the distance between oneself and others  Stop shaking hands or hugging  Wear a mask / face covering  Hand washing  Disinfect and/or clean objects and surfaces |
|  | **Informed**: corresponds to those who selected 5 out of 6 answers |  |
|  | **A little informed**: corresponds to those who selected 4 out of these 7 options | Reduce contact with others by avoiding crowds, staying at home, etc  Increase the distance between oneself and others  Stop shaking hands or hugging  Wear a mask / face covering  Hand washing  Disinfect and/or clean objects and surfaces  Wear gloves (even though literature was clear on the use of gloves, at the time it was not at all clear for the general public). |
|  | **Not at all informed**: corresponds to those who selected any of these options | Other  Do not know / prefer not to respond  Praying |

# Results

## Respondents’ profile

Table S5: Descriptive statistics of household survey respondents by study site

| **Weighted mean (SD) or N (weighted %)** | **Bangladesh (N = 842)** | **CAR (N = 1,045)** | **DRC (N = 657)** | **p-value** |
| --- | --- | --- | --- | --- |
| **Sex, N (weighted %)** |  |  |  | **<0.001** |
| Men | 443 (52.55) | 409 (37.28) | 219 (33.33) |  |
| Women | 398 (47.32) | 636 (62.72) | 438 (66.67) |  |
| Other | 1 (0.13) | 0 (0) | 0 (0) |  |
| **Age, years, N (weighted %)** |  |  |  | **<0.001** |
| 18 - 29 | 249 (29.19) | 271 (26.54) | 263 (40.03) |  |
| 30-59 | 545 (65.13) | 662 (61.43) | 327 (49.77) |  |
| 60+ | 48 (5.68) | 112 (12.03) | 67 (10.20) |  |
| **Setting, N (weighted %)** |  |  |  | **<0.001** |
| Urban | 687 (81.90) | 652 (62.39) | 137 (20.85) |  |
| Rural | 155 (18.10) | 393 (37.61) | 520 (79.15) |  |
| **Professions, N (weighted %) (Bangladesh)** |  |  |  |  |
| Daily wage worker | 127 (15.04) |  |  |  |
| Housewife | 388 (45.96) |  |  |  |
| Business owner | 116 (13.73) |  |  |  |
| Private sector | 75 (9.01) |  |  |  |
| Student | 32 (4.10) |  |  |  |
| Other | 29 (3.45) |  |  |  |
| None | 42 (4.83) |  |  |  |
| N/A | 33 (3.87) |  |  |  |
| **Professions, N (weighted %) (CAR & DRC)** |  |  |  | **<0.001** |
| Farming |  | 463 (24.24) | 514 (78.23) |  |
| Trade |  | 399 (50.02) | 53 (8.07) |  |
| Public official |  | 41 (6.42) | 29 (4.41) |  |
| Other |  | 63 (9.28) | 25 (3.81) |  |
| None |  | 79 (10.03) | 36 (5.48) |  |
| **Religion, N (weighted %) (CAR & DRC)** |  |  |  | **<0.001** |
| Christian |  | 961 (87.53) | 651 (99.09) |  |
| Muslim |  | 35 (6.35) | 1 (0.15) |  |
| Animist |  | 48 (6.10) | 1 (0.15) |  |
| Other |  | 1 (0.02) | 2 (0.30) |  |
| N/A |  | 0 (0) | 2 (0.30) |  |
| **Upazila, N (weighted %) (Bangladesh)** |  |  |  |  |
| Chakaria | 166 (19.40) |  |  |  |
| Cox's Bazaar | 170 (20.18) |  |  |  |
| Kutubdia | 37 (4.30) |  |  |  |
| Maheshkhali | 101 (12.15) |  |  |  |
| Pekua | 62 (7.39) |  |  |  |
| Ramu | 89 (10.63) |  |  |  |
| Teknaf | 114 (13.73) |  |  |  |
| Ukhia | 103 (12.22) |  |  |  |
| **District, N (weighted %) (CAR)** |  |  |  |  |
| Begoua |  | 145 (3.58) |  |  |
| Bimbo |  | 444 (27.92) |  |  |
| Bangui |  | 456 (68.51) |  |  |
| **District, N (weighted %) (DRC)** |  |  |  |  |
| Bibwe |  |  | 24 (3.65) |  |
| Busumba |  |  | 42 (6.39) |  |
| Butare Centre |  |  | 23 (3.50) |  |
| Butumbalong |  |  | 37 (5.63) |  |
| Bweru |  |  | 32 (4.87) |  |
| Kabati |  |  | 32 (4.87) |  |
| Kalengere |  |  | 36 (5.48) |  |
| Kamonyi |  |  | 50 (7.61) |  |
| Kirumbu |  |  | 44 (6.70) |  |
| Kitabi |  |  | 26 (3.96) |  |
| Kitso |  |  | 28 (4.26) |  |
| Kivuye |  |  | 23 (3.50) |  |
| Mbuhi |  |  | 40 (6.09) |  |
| Mohoto |  |  | 23 (3.50) |  |
| Muhongozi |  |  | 24 (3.65) |  |
| Mweso |  |  | 137 (20.85) |  |
| Rugarama |  |  | 25 (3.81) |  |
| Rugogwe |  |  | 11 (1.67) |  |
| **Education, N (weighted %)** |  |  |  | **<0.001** |
| None | 170 (20.00) | 136 (10.93) | 299 (45.51) |  |
| Primary | 315 (37.18) | 461 (35.60) | 198 (30.14) |  |
| Secondary | 272 (32.54) | 405 (46.41) | 150 (22.83) |  |
| Tertiary | 83 (10.07) | 43 (7.06) | 10 (1.52) |  |
| N/A | 2 (0.21) | 0 (0) | 0 (0) |  |
