# Supplementary material for: “Everything had stopped, no meeting, no gathering”: Social interactions during the COVID-19 pandemic in the Central African Republic, the Democratic Republic of Congo, and Bangladesh
Source: PLoS One. 2025 May 27;20(5):e0323108. doi: 10.1371/journal.pone.0323108 (PMC12112413; doi:10.1371/journal.pone.0323108)
Supplement: Attachment — Submitted filename: Replies to journal and reviewers comments_Feb 5 2025.docx [file pone.0323108.s004.docx]

February 5, 2025

**Response to the journal and reviewers’ comments on the article:**

**“Everything had stopped, no meeting, no gathering”: social interactions during the COVID-19 pandemic in the Central African Republic, the Democratic Republic of Congo, and Bangladesh.**

Dear Editors of Plos One,

Please find below our point-by-point response to the editorial and reviewers’ comments.

We have indicated whether and how we have revised the text and referenced line numbers in the track change version of the revised paper with author details.

We remain at your disposal for any clarification, and we look forward to hearing from you.

With thanks,

Chiara Altare

**_______**

**Journal Requirements:**

**1. When submitting your revision, we need you to address these additional requirements.**

Please ensure that your manuscript meets PLOS ONE's style requirements, including those for file naming. The PLOS ONE style templates can be found at

<https://journals.plos.org/plosone/s/file?id=wjVg/PLOSOne_formatting_sample_main_body.pdf>

and 
<https://journals.plos.org/plosone/s/file?id=ba62/PLOSOne_formatting_sample_title_authors_affiliations.pdf>

**Authors’ reply:** Thank you. We revised the guidelines and updated the paper accordingly

**2. Please include a complete copy of PLOS’ questionnaire on inclusivity in global research in your revised manuscript.** Our policy for research in this area aims to improve transparency in the reporting of research performed outside of researchers’ own country or community. The policy applies to researchers who have travelled to a different country to conduct research, research with Indigenous populations or their lands, and research on cultural artefacts. The questionnaire can also be requested at the journal’s discretion for any other submissions, even if these conditions are not met. Please find more information on the policy and a link to download a blank copy of the questionnaire here: <https://journals.plos.org/plosone/s/best-practices-in-research-reporting>. Please upload a completed version of your questionnaire as Supporting Information when you resubmit your manuscript.

**Authors’ reply:** Thank you. We added the questionnaire as Supporting Information.

**3. In the ethics statement in the Methods, you have specified that verbal consent was obtained. Please provide additional details regarding how this consent was documented and witnessed, and state whether this was approved by the IRB.**

**Authors’ reply:** Yes, verbal consent was approved by JHSPH IRB as well as in country IRBs. Consent was documented on the data collection tool (i.e. interviewer could not proceed to the interview without confirming consent was given).

**4. Thank you for stating in your Funding Statement:**

*This work was supported by the Bureau for Humanitarian Assistance, US Agency for International Development (*[*https://www.usaid.gov/*](https://www.usaid.gov/)*) under Grant Number 720FDA20GR00228. The funders had no role in study design, data collection and analysis, decision to publish, or preparation of the manuscript.*

**Please provide an amended statement that declares *all* the funding or sources of support** (whether external or internal to your organization) received during this study, as detailed online in our guide for authors at <http://journals.plos.org/plosone/s/submit-now>. Please also include the statement “There was no additional external funding received for this study.” in your updated Funding Statement.

Please include your amended Funding Statement within your cover letter. We will change the online submission form on your behalf.

**Authors’ reply:** Please find here the updated funding statement. Thanks for editing it in the online submission form.

*This work was supported by the Bureau for Humanitarian Assistance, US Agency for International Development (*[*https://www.usaid.gov/*](https://www.usaid.gov/)*) under Grant Number 720FDA20GR00228. The funders had no role in study design, data collection and analysis, decision to publish, or preparation of the manuscript. There was no additional external funding received for this study.*

**5. Thank you for stating the following in the Acknowledgments Section of your manuscript:** 
*We would like to thank the United States Bureau for Humanitarian Assistance (USAID/BHA) for funding this work; Marissa Smith, Madison Bates, Natalia Hernandez Morfin, and Sharon Leslie helped analyze the data and draft the case study reports.*

*Group authorships are as follows: 
IMPACT CAR Team: Ugo Semat, Melissa Pointet,  Sarah McArthur, Samuel Carcanague, Ana Tolosa, Rodrigue Biguioh; IMPACT DRC Team: Roxana Mullafiroze, Jasper Linke, Olivier Cecchi, Nayana Das, Katie Rickard; Jean-Paul Mushamalirwa, Destin Ruhinda, Nadia Lehmann, Marie Amandine, Eliora Henzler, Audrey Gallecier, Benoit Besnardeau, Noortje Gerritsma; IMPACT Bangladesh Team: Roxana Mullafiroze, Jasper Linke, Olivier Cecchi, Nayana Das, Katie Rickard, Rosie Witton, Sara Chowdhury, Zia Foisal, Teresa Schwarz, Glenn Poresh.*

We note that you have provided funding information that is not currently declared in your Funding Statement. However, funding information should not appear in the Acknowledgments section or other areas of your manuscript. We will only publish funding information present in the Funding Statement section of the online submission form.

**Please remove any funding-related text from the manuscript and let us know how you would like to update your Funding Statement.** Currently, your Funding Statement reads as follows:

This work was supported by the Bureau for Humanitarian Assistance, US Agency for International Development (<https://www.usaid.gov/>) under Grant Number 720FDA20GR00228. The funders had no role in study design, data collection and analysis, decision to publish, or preparation of the manuscript.

Please include your amended statements within your cover letter; we will change the online submission form on your behalf.

**Authors’ reply:** Noted, please find here the updated funding statement (same as in response to point 4). Thanks for editing it in the online submission form.

*This work was supported by the Bureau for Humanitarian Assistance, US Agency for International Development (*[*https://www.usaid.gov/*](https://www.usaid.gov/)*) under Grant Number 720FDA20GR00228. The funders had no role in study design, data collection and analysis, decision to publish, or preparation of the manuscript. There was no additional external funding received for this study.*

6. One of the noted authors is a **group or consortium** "IMPACT CAR Team; IMPACT DRC Team; IMPACT Bangladesh Team". In addition to naming the author group, **please list the individual authors and affiliations within this group in the acknowledgments section of your manuscript. Please also indicate clearly a lead author for this group along with a contact email address.**

**Authors’ reply:** Please find here the revised Acknowledgements (also added in the revised version of the manuscript):

*****

We would like to thank Marissa Smith, Madison Bates, Natalia Hernandez Morfin, and Sharon Leslie who helped analyze the data and draft the case study reports.

Group authorships are as follows: 
**IMPACT CAR Team**

Affiliation: IMPACT CAR office

Lead author: Melissa Pointet, Samuel Carcanague

Contact email: Katie Rickard [katie.rickard@impact-initiatives.org](mailto:katie.rickard@impact-initiatives.org)

Group members: Ugo Semat, Melissa Pointet, Sarah McArthur, Samuel Carcanague, Ana Tolosa, Rodrigue Biguioh. Katie Rickard, Roxana Mullafiroze

**IMPACT DRC Team**

Affiliation: IMPACT DRC office

Lead author: Jasper Linke, Noortje Gerritsma

Contact email: Katie Rickard [katie.rickard@impact-initiatives.org](mailto:katie.rickard@impact-initiatives.org)

Group members: Roxana Mullafiroze, Jasper Linke, Olivier Cecchi, Nayana Das, Katie Rickard; Jean-Paul Mushamalirwa, Destin Ruhinda, Nadia Lehmann, Marie Amandine, Eliora Henzler, Audrey Gallecier, Benoit Besnardeau, Noortje Gerritsma.

**IMPACT Bangladesh Team**

Affiliation: IMPACT Bangladesh office

Lead author: Sara Chowdhury

Contact email Katie Rickard [katie.rickard@impact-initiatives.org](mailto:katie.rickard@impact-initiatives.org)

Group members: Roxana Mullafiroze, Jasper Linke, Olivier Cecchi, Nayana Das, Katie Rickard, Rosie Witton, Sara Chowdhury, Zia Foisal, Teresa Schwarz, Glenn Poresh.

*****

7. We note that there is identifying data in the Supporting Information file "Supplementary material_Soc Interactions_July 3 24". Due to the inclusion of these potentially identifying data, we have removed this file from your file inventory. Prior to sharing human research participant data, authors should consult with an ethics committee to ensure data are shared in accordance with participant consent and all applicable local laws.

Data sharing should never compromise participant privacy. It is therefore not appropriate to publicly share personally identifiable data on human research participants. The following are examples of data that should not be shared:

- Name, initials, physical address
- Ages more specific than whole numbers
- Internet protocol (IP) address
- Specific dates (birth dates, death dates, examination dates, etc.)
- Contact information such as phone number or email address
- Location data
- ID numbers that seem specific (long numbers, include initials, titled “Hospital ID”) rather than random (small numbers in numerical order)

Data that are not directly identifying may also be inappropriate to share, as in combination they can become identifying. For example, data collected from a small group of participants, vulnerable populations, or private groups should not be shared if they involve indirect identifiers (such as sex, ethnicity, location, etc.) that may risk the identification of study participants.

Additional guidance on preparing raw data for publication can be found in our Data Policy (<https://journals.plos.org/plosone/s/data-availability#loc-human-research-participant-data-and-other-sensitive-data>) and in the following article: <http://www.bmj.com/content/340/bmj.c181.long>.

**Please remove or anonymize all personal information** (<specific identifying information in file to be removed>), ensure that the data shared are in accordance with participant consent, and re-upload a fully anonymized data set. Please note that spreadsheet columns with personal information must be removed and not hidden as all hidden columns will appear in the published file.

**Authors’ reply:** We have submitted a revised version of supplementary material.

**8. Please include captions for your Supporting Information files at the end of your manuscript, and update any in-text citations to match accordingly**. Please see our Supporting Information guidelines for more information: <http://journals.plos.org/plosone/s/supporting-information>.

**Authors’ reply:** Noted. We added captions for the supporting information at the end of the manuscript.

**Reviewer's Responses to Questions**

**Comments to the Author**

1. Is the manuscript technically sound, and do the data support the conclusions?

The manuscript must describe a technically sound piece of scientific research with data that supports the conclusions. Experiments must have been conducted rigorously, with appropriate controls, replication, and sample sizes. The conclusions must be drawn appropriately based on the data presented.

Reviewer #1: Yes

Reviewer #2: Yes

2. Has the statistical analysis been performed appropriately and rigorously?

Reviewer #1: No

Reviewer #2: Yes

3. Have the authors made all data underlying the findings in their manuscript fully available?

The [PLOS Data policy](https://nam02.safelinks.protection.outlook.com/?url=http://track.editorialmanager.com/CL0/http:%2F%2Fwww.plosone.org%2Fstatic%2Fpolicies.action%23sharing/1/010f0193f6db1ddc-0cbab1ce-c8b6-470a-9104-ce54b59d8af9-000000/3Kmfcs2ID2UEjnSa_7tQsMkZf1JuwFnd1Hkw3nU3d9E=190&data=05|02|chiara.altare@jhu.edu|ba2f6bfced2a4158e31508dd23d0c84a|9fa4f438b1e6473b803f86f8aedf0dec|0|0|638706102199578011|Unknown|TWFpbGZsb3d8eyJFbXB0eU1hcGkiOnRydWUsIlYiOiIwLjAuMDAwMCIsIlAiOiJXaW4zMiIsIkFOIjoiTWFpbCIsIldUIjoyfQ==|0|||&sdata=bK62I731iyQ7Na0VOybmYv81DD70ENYIFGcltvr5Grc=&reserved=0) requires authors to make all data underlying the findings described in their manuscript fully available without restriction, with rare exception (please refer to the Data Availability Statement in the manuscript PDF file). The data should be provided as part of the manuscript or its supporting information, or deposited to a public repository. For example, in addition to summary statistics, the data points behind means, medians and variance measures should be available. If there are restrictions on publicly sharing data—e.g. participant privacy or use of data from a third party—those must be specified.

Reviewer #1: Yes

Reviewer #2: Yes

4. Is the manuscript presented in an intelligible fashion and written in standard English?

PLOS ONE does not copyedit accepted manuscripts, so the language in submitted articles must be clear, correct, and unambiguous. Any typographical or grammatical errors should be corrected at revision, so please note any specific errors here.

Reviewer #1: Yes

Reviewer #2: Yes

**5. Review Comments to the Author**

Please use the space provided to explain your answers to the questions above. You may also include additional comments for the author, including concerns about dual publication, research ethics, or publication ethics. (Please upload your review as an attachment if it exceeds 20,000 characters)

**Reviewer #1:** The paper presents a valuable investigation into how social interactions in three humanitarian settings (Democratic Republic of the Congo, Central African Republic, and Cox’s Bazar, Bangladesh) changed during the first year of the COVID-19 pandemic. This is a significant contribution given the unique challenges and vulnerabilities these settings face.

One of the novel aspects is the comparative mixed-methods approach, combining representative household surveys with qualitative data from focus group discussions and key informant interviews. This methodology provides a comprehensive understanding of social dynamics and preventive behaviors during an epidemic.

The paper highlights the discrepancy between knowledge about and practice of preventive behaviors and identifies factors associated with non-compliant behaviors, such as reluctance towards COVID-19 vaccination and living conditions in IDP communities.

**Authors’ reply:** Thank you.

**Strengths:**
The study addresses a critical gap in understanding social interactions in humanitarian settings during a pandemic, which has been underexplored in existing literature.

The methodology is robust, with a clear explanation of the mixed-methods approach and the rationale for selecting the study sites. The use of both quantitative and qualitative data enriches the analysis.
The findings are relevant and timely, providing insights that could inform future public health interventions in similar settings. For example, the identification of trust in authorities and socio-economic factors as drivers of behavior is crucial for designing effective public health strategies. The paper is well-organized, with a clear structure that guides the reader through the background, methodology, results, and discussion.

**Authors’ reply:** Thank you.

**Weaknesses:**
The study's reliance on self-reported data could introduce bias, particularly in sensitive topics such as compliance with preventive measures and vaccination attitudes. **The paper does not adequately address how this potential bias was mitigated. The paper could benefit from a more in-depth discussion of the limitations of the study.** For example, the authors should discuss the **potential impact of the non-reachable settlements in DRC on the representativeness of the data**.

**Authors’ reply:** Thank you for raising this point. We have expanded the limitations of the paper highlighting possible biases affecting the study (lines 456 to 465).

**Some of the statistical analyses, such as the multivariate logistic regression, are presented without sufficient detail on the assumptions and checks performed to validate the models.**

**Authors’ reply:** Thank you for your comment. We clarified in the method section (lines 227 to 239) that all variables used in the analysis are categorical as we estimated a logistic regression. We also clarified that we did not need to test for linearity, normality and equal variance assumptions as all independent and dependent variables were categorical.

Following your comment, we added a sentence about the use of the Wald test to the logistic regression models to test the global null hypothesis and assess the model validity (line 242). Results from all regression models showed that global null hypothesis could be rejected, (p-value<0.001) indicating that the independent variables included in these models improve the predictability of the results (lines 298-299).

**Suggestions for Improvement:**

Address the potential biases introduced by self-reported data by discussing any steps taken to validate the responses or by **including a section on the limitations of self-reported data**. For example, cross-referencing self-reported behaviors with observed behaviors or using triangulation methods could strengthen the validity of the findings. **Expand the discussion on the limitations of the study**, particularly regarding the non-reachable settlements in DRC and any other logistical challenges encountered. This transparency will help readers critically assess the robustness of the study.

**Authors’ reply:** Thank you for this suggestion. We expanded the limitation section in the discussion (lines 452 to 473).

**Provide more detail on the statistical analysis methods, including the assumptions of the logistic regression models and how these were checked**. **This could include information on multicollinearity tests, goodness-of-fit measures, and any sensitivity analyses performed.**

**Authors’ reply:** Thank you for this suggestions. We made edits in the methods sections (lines 227 to 239) and clarified the type of variables used in the analysis and the assumptions that needed to the be tested.

**Enhance the discussion on the implications of the findings for public health policy and practice**. For example, the paper could suggest specific interventions to increase trust in authorities or address the socio-economic factors influencing preventive behaviors.

**Authors’ reply:** Thank you for your suggestion. We discuss implications for public health policy and practice related to: i) the location of interaction (page 22): our findings show that most interactions occurred at home, pointing to the need of a mix of measures (at both individual and societal levels); ii) barriers to adopting preventive measures (page 22): we highlight the importance of identifying why the uptake of a certain measure is low in order to design strategies that are contextualized; iii) trust in authorities (page 23): we highlighted how targeted sensitization and information campaigns should address fears and questions of communities; iv) increase trust in vaccination: concrete strategies to increase trust in vaccinations are listed on page 23-24.

**Reviewer #2:** The study is very important and touches a very critical aspect of population health during pandemic. Please consider the following:

**1- Adjust the article and use the past tense.**

**Authors’ reply:** Thank you. We revised the present tense where it was not correct.

**2- Add to the keywords list: pandemic; population health.**

**Authors’ reply:** Thank you for your suggestions. We added the keywords you proposed.

**3- Shed light on the impact of pandemic and borders closure on health system and supply chains.**

**Authors’ reply:** While we agree that the impact of the pandemic and border closure on the health system and supply chain was serious, we think it is outside the scope of this specific paper as we focus on social interactions and factors facilitating or hindering compliance with recommended behaviors.

**4- Identify the impact of humanitarian aids supported underserved and vulnerable population.**

**Authors’ reply:** Similarly as above, while we would welcome clarification on the comment, we think discussing the humanitarian aid provided to affected communities is outside the scope of the paper as it focuses on social interactions during the COVID-19 pandemic.

**5- What are the common characteristics and differences between the three countries? and define the rationale of selecting those countries.**

**Authors’ reply:** Thank you for your comment. We provided more details about why the three countries were selected at the beginning of the method section (lines 125-127).

The goal of the larger multi-country project was to investigate the COVID-19 epidemiology, health service utilization, COVID-19 knowledge and social interactions, and health care seeking behavior in different humanitarian settings. We therefore chose countries affected by large scale humanitarian crises, from different regions, and where the study partners had ongoing operations.

**6- Shed light on the interaction between pandemic related psychological dilemma and population dynamic.**

**Authors’ reply:** Thank you. We added a paragraphs at the end of the discussion reflecting the way we understood the comment (lines 447-451).

**7- Qualitative analysis is inadequate.**

**Authors’ reply:** Thank you. We would appreciate some clarifications to the comment. We have added details about how the qualitative analysis was conducted in the method (lines 217-220).

**8- In contrary to other population, explain the reasons for higher rates of covid-19 vaccination acceptance.**

**Authors’ reply:** Willingness to be vaccinated against COVID-19 was very high in Bangladesh (98%), but less pronounced in CAR (“certainly”: 32%) and even less in DRC (“certainly” 17%). We argue that willingness to be vaccinated is influenced by the trust in authorities and their policies. In DRC, this low trust in authorities could stem from decades of conflict and misalignment with national authorities. The response to the 10^th^ Ebola outbreak (2018-2020) deeply reduced trust and had longer term consequences on vaccine acceptance.

**9- Add a section for limitation and recommendations.**

**Authors’ reply:** Thank you. We expanded the limitation sections (lines 452 to 473) and we highlighted a few recommendations in the conclusion (lines 476 to 496).

6. PLOS authors have the option to publish the peer review history of their article ([what does this mean?](https://nam02.safelinks.protection.outlook.com/?url=https://track.editorialmanager.com/CL0/https:%2F%2Fjournals.plos.org%2Fplosone%2Fs%2Feditorial-and-peer-review-process%23loc-peer-review-history/1/010f0193f6db1ddc-0cbab1ce-c8b6-470a-9104-ce54b59d8af9-000000/3KVEvUYV_uu5jeoH5hwfYg9-D9fwZsjgcAMFtGAcjVo=190&data=05|02|chiara.altare@jhu.edu|ba2f6bfced2a4158e31508dd23d0c84a|9fa4f438b1e6473b803f86f8aedf0dec|0|0|638706102199592475|Unknown|TWFpbGZsb3d8eyJFbXB0eU1hcGkiOnRydWUsIlYiOiIwLjAuMDAwMCIsIlAiOiJXaW4zMiIsIkFOIjoiTWFpbCIsIldUIjoyfQ==|0|||&sdata=90fowUtN7ywRIgeHpCJS7l+/1zaQuk4mM2bJazQ3ptg=&reserved=0)). If published, this will include your full peer review and any attached files.

If you choose “no”, your identity will remain anonymous but your review may still be made public.

**Do you want your identity to be public for this peer review?** For information about this choice, including consent withdrawal, please see our [Privacy Policy](https://nam02.safelinks.protection.outlook.com/?url=https://track.editorialmanager.com/CL0/https:%2F%2Fwww.plos.org%2Fprivacy-policy/1/010f0193f6db1ddc-0cbab1ce-c8b6-470a-9104-ce54b59d8af9-000000/yDHTOBwU-bBawwCFThRcO9KZDrSrTOkRvrSxII69ilc=190&data=05|02|chiara.altare@jhu.edu|ba2f6bfced2a4158e31508dd23d0c84a|9fa4f438b1e6473b803f86f8aedf0dec|0|0|638706102199607225|Unknown|TWFpbGZsb3d8eyJFbXB0eU1hcGkiOnRydWUsIlYiOiIwLjAuMDAwMCIsIlAiOiJXaW4zMiIsIkFOIjoiTWFpbCIsIldUIjoyfQ==|0|||&sdata=EklH3eyLEHyRANM/YIDUXgiU8v+/52TsNItMWRjuOA0=&reserved=0).

Reviewer #1: **Yes:** Olivier Mukuku

Reviewer #2: **Yes:** WEAM BANJAR
