# Supplementary material for: “Everything had stopped, no meeting, no gathering”: Social interactions during the COVID-19 pandemic in the Central African Republic, the Democratic Republic of Congo, and Bangladesh
Source: PLoS One. 2025 May 27;20(5):e0323108. doi: 10.1371/journal.pone.0323108 (PMC12112413; doi:10.1371/journal.pone.0323108)
Supplement: Attachment — Submitted filename: Responses to comments-March 28 2025.docx [file pone.0323108.s005.docx]

Baltimore March 28, 2025

**Response to the journal and reviewers’ comments on the article:**

**“Everything had stopped, no meeting, no gathering”: social interactions during the COVID-19 pandemic in the Central African Republic, the Democratic Republic of Congo, and Bangladesh.**

Dear Editors of Plos One,

Please find below our point-by-point response to the editorial and reviewers’ comments.

We have indicated whether and how we have revised the text and referenced line numbers in the track change version of the revised paper with author details.

We remain at your disposal for any clarification, and we look forward to hearing from you.

With thanks,

Chiara Altare

_______

**Reviewer’s comments**

1. This article, according to the authors, attempted to understand the spread of COVID-19 in humanitarian settings. However, it was not clear on what considerations the Cox's Bazar area of Bangladesh was selected as a site or an area with a humanitarian context! . The Cox's Bazar area of Bangladesh does not meet the criteria to be called an area with a humanitarian crisis just because it is hosting Rohingya refugees. According to the authors, the site was selected for this study as it is the location of the IMPACT project and possibly the site of a larger multi-country project. It is important, therefore, to provide adequate justifications under the methodology section on the selection of Cox's Bazar as a site that is comparable to CAR and the DRC in terms of demographic, socio-economic, and humanitarian characteristics.

**Authors’ reply:** Thank you for your comment. We agree that Cox’s Bazar is not a humanitarian emergency simply because it is hosting Rohingya refugees. However, we believe it can be considered a fragile setting given pre-existing vulnerabilities that were exacerbated by the rapid influx of refugees. We attempted to make this differentiation by using “humanitarian and fragile settings” throughout the document. We did notice a few spots where we only referenced it as a humanitarian setting, and we corrected this.

We also edited the methodology as requested clarifying the differences across study sites [lines 116 – 120].

2. In the analysis section, it has been mentioned that "thematic analysis was applied to summarize relevant themes across study participants as well as to identify differences across study sites."

In the result section of the qualitative data, no such analysis by thematic areas and comparison by different sites were noted. It is important that the analyzed qualitative data are presented by the main thematic areas of the study, and a summary of the main findings is presented in a way that shows comparison or commonalities between the sites.

**Authors’ reply:** Thank you for the comment. We revised the qualitative results section (page 18 to 21) to highlight the following:

- The themes that were identified in the analysis are: Attitude and knowledge about COVID-19, knowledge and practice of preventative measures, perceptions about vaccines, information sources, and changes in social interactions.
- We made reference to the longer version of the results by theme available in the three country reports.
- We reorganized the findings in the paper around the above mentioned themes but in a more succinct way.

3. More description should be provided on how the qualitative data were analyzed. Such as how the data were transcribed, which software was used to record, and code and for transcription of the data. How the consistency and accuracy were checked as the interviews and FGDs were done in three sites in the local language.

**Authors’ reply:** Thank you for the comment. We provided more details how qualitative data were managed and analyzed on page 10 [Lines 219-225].

4. It is also important to highlight in the section on strength and limitation if the translation of the KII and FGD into English from the local language introduced any inconsistencies and how they were checked and prevented.

**Authors’ reply:** Thank you. We clarified that KII and FGD notes were directly taken in English / French by the interviewer and note takers, as deemed easier [lines 171-173]. Therefore, we did not have to translate them again, as the core research team could work in both languages.

5. For sample size selection, a "prevalence of 50%" was used to select the household number for Cox's Bazar and CAR. It is not clear what this "50% prevalence" refers to. What is the source of such an assumption? The pandemic was a new event. Therefore, what was the reference underlying the author's decision to use a "50% prevalence" for what variable in order to select the right sample? Please provide some clarity in the text to make the readers understand the rationale behind such an assumption.

**Authors’ reply:** To calculate the sample size required for a survey, you usually need to define three key parameters: “prevalence of the variable of interest” (i.e. the population’s proportion who would have a certain characteristics or knowledge or behavior), margin of error and confidence level. As in our case we had multiple variables of interest for which no previous estimate was known, we took the conservative approach of 50% that would generate the biggest sample size. See: <https://www.calculator.net/sample-size-calculator.html>

We changed “prevalence” to population’s proportion with variable of interest in the method section.

6. In Table 2, please provide the mean age (SD) or median (IR) age of the respondents by three study sites. It is also important to present the  gender characteristics of the respondents though these are availabel in the supplementary table.

**Authors’ reply:** We have added information about age and gender characteristics to Table 2. We have also provided the mean and standard deviation of age by region in Results section (lines 271 – 272): “Mean and SD of respondent age in years were 36.70 (SD 11.84) in Bangladesh, 40.01 (SD 13.99) in CAR, and 35.62 (SD 14.40) in DRC.”

7. In Tables 3 and 4, we understand that the data are analyzed using a multivariate logistic regression analysis. As such, the Odds Ratios presented in these tables should be named as adjusted Odds Ratios (aOR) unless the authors have other justifications for not naming these ratios as "aOR".

**Authors’ reply:** Thank you, we have changed OR to aOR in tables 3 and 4 and in the narrative results page 15.

8. It is not clear why (in Table 3) the columns for "Displacement" for Bangladesh and CAR are empty. No displaced people from either of these two countries were included in the sample.? Please provide a justification in the footnote of the table.

**Authors’ reply:** In Bangladesh, no information about displacement was investigated during the survey as all respondents were from the host/ resident communities. In CAR, all individuals who were displaced (N = 190) were residing in urban area. Therefore, the displacement category in CAR coincided with the urban category. We were therefore unable to provide aOR of displacement in multivariate regression model.

9. The author's findings that "IDPs were at higher risk and increased and faced increased barriers to protecting themselves from COVID-19" is not substantiated by data presented in Table 4 (P value not significant). Any clarity will be helpful.

**Authors’ reply:** Thank you for raising this point. Results in table 3 do support the statement that displacement was a risk factor for increasing the frequency of meetings, however you are right that displacement is not statically significant in table 4 (i.e. we could not identify it as a risk factor for increased duration of meetings). We edited the conclusion accordingly [lines 736-738].
